# Multiparametric 18F–FDG PET/MR follow-up in a patient with autoimmune pancreatitis

**DOI:** 10.1186/s41824-017-0016-9

**Published:** 2017-11-22

**Authors:** Isabel Rauscher, Matthias Eiber, Hana Algül, Jens T. Siveke, Gregor Weirich, Anna M. Schlitter, Ambros J. Beer

**Affiliations:** Technical University of Munich Klinikum rechts der Isar, Department of Nuclear Medicine, Munich, Germany

## Abstract

**Background:**

Positron emission tomography/magnetic resonance imaging (PET/MR) is a new multimodal imaging technique, which might improve the diagnostic performance not only in oncological patients but also in patients with non-neoplastic inflammatory lesions as routinely used ^18^F-FDG is not a cancer specific agent.

**Case Presentation:**

Multiparametric ^18^F–FDG PET/MR in a woman with pain in the upper abdomen and inconclusive laboratory and clinical data presenting with moderately increased, diffuse ^18^F–FDG uptake with delayed contrast enhancement, diffusion-restriction and focal enlargement in the pancreatic body being suggestive for autoimmune pancreatitis (AIP). Follow-up ^18^F–FDG PET/MR after initiation of steroid therapy confirmed complete resolution of imaging abnormalities.

**Conclusion:**

^18^F–FDG PET/MR might be valuable in the management of AIP providing complementary data regarding accurate diagnosis and monitoring therapy response to avoid unnecessary surgery.

## Background

Recently, positron emission tomography/magnetic resonance imaging (PET/MR) has been introduced as a new multimodal imaging technique combining morphological and functional information. For the diagnosis of pancreatic cancer, fluorine-18 fluorodeoxyglucose (^18^F–FDG)-PET has been most widely used (Rijkers et al., 2014). However, ^18^F–FDG is not a cancer specific agent and its uptake has been described in non-neoplastic inflammatory lesions as well (Ozaki et al., 2008).

## Case Presentation

**Fig. 1:**
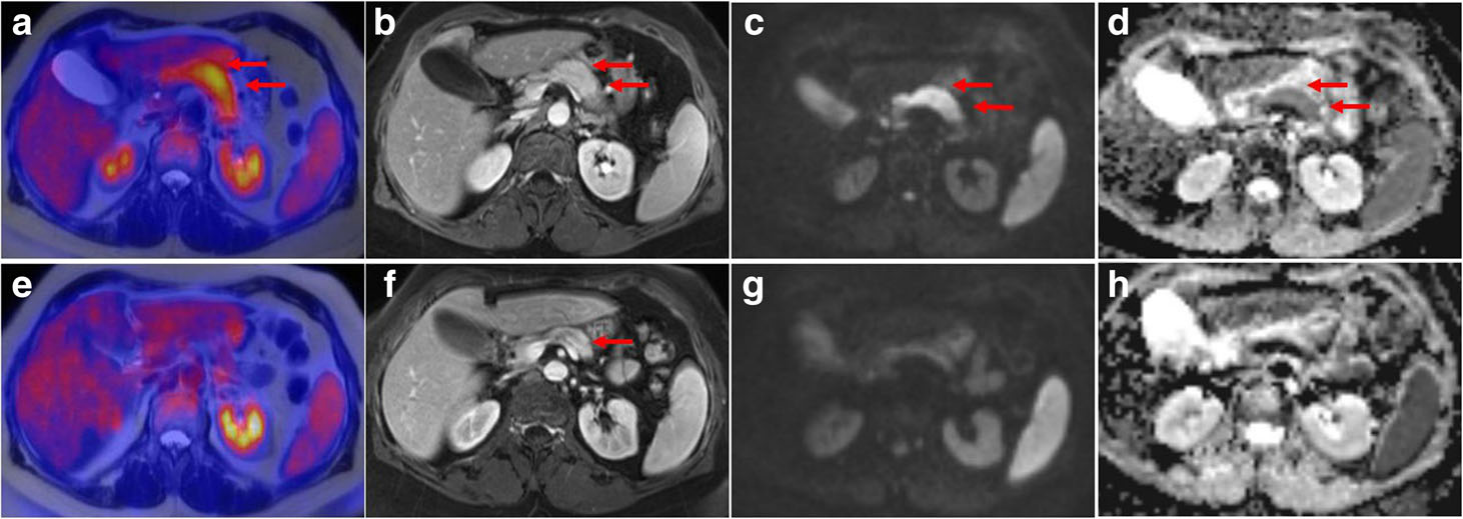
Multiparametric ^18^F-FDG PET/MR in a patient with autoimmune pancreatitis and follow-up ^18^F-FDG PET/MR after initiation of steroid therapy

We present the case of a 58-year-old female with pain in the left upper abdomen since 2 months in the absence of other clinical symptoms. Laboratory data were inconclusive and a history of alcohol abuse could be excluded. Endoscopic ultrasound and CT ruled out choledocholithiasis, but revealed a bulky pancreas indicating inflammation. ^18^F–FDG PET/MR was performed to rule out possible unrecognized pancreatic cancer (Fig. 1). The scan demonstrated moderately increased, diffuse ^18^F–FDG uptake in the pancreatic body (A, arrow) with corresponding MRI showing enlargement of the pancreatic body with delayed contrast enhancement in T1 VIBE sequence (B, arrow), high diffusion-weighted imaging (DWI) signal (C; b value = 400) and low ADC values (D) being suggestive for autoimmune pancreatitis (AIP) as described before (Kamisawa et al., 2010; Lee & Sahani, 2014). Subsequently endoscopic ultrasound-guided biopsy of the pancreas excluded pancreatic cancer and confirmed chronic pancreatic inflammation. In general, AIP is responsive to corticosteroid therapy (Lee & Sahani, 2014; Kamisawa et al., 2009). Follow-up ^18^F–FDG PET/MR 4 weeks after initiation of steroid therapy confirmed complete resolution of imaging abnormalities with normalization of ^18^F–FDG uptake (E), size reduction of affected pancreatic parenchyma with normalization of pancreatic enhancement (F) and diffusion restriction (G and H).

## Discussion

Along with clinical, laboratory, and histopathological data, imaging plays an important role in the diagnosis and management of AIP, however, imaging appearance of AIP varies widely. Certain nonspecific imaging features in cross-sectional imaging have been defined which are more likely to represent AIP than alternate diagnoses (e.g. pancreatic cancer) (Lee & Sahani, 2014). Recent studies suggest that ^18^F–FDG PET may prove valuable in providing complementary data in delineating the extent of organ involvement and staging the extent of disease as extra-pancreatic sites of involvement are also often seen in AIP (Lee & Sahani, 2014). Further, additional ^18^F–FDG PET might help guiding biopsy early in the diagnostic evaluation, and monitoring response to therapy.

## Conclusion

In complex cases, when it’s important to gather all available functional and morphological information to exclude malignancy, combined ^18^F–FDG PET/MR may prove valuable in the management of AIP regarding accurate diagnosis and monitoring therapy response to avoid unnecessary surgery.
